# Viral co‐infections are associated with increased rates of hospitalization in those with influenza

**DOI:** 10.1111/irv.12967

**Published:** 2022-03-18

**Authors:** Kerry L. Shannon, Valerie O. Osula, Kathryn Shaw‐Saliba, Justin Hardick, Breana McBryde, Andrea Dugas, Yu‐Hsiang Hsieh, Bhakti Hansoti, Richard E. Rothman, Emergency Department National Influenza Network Investigators, Mark Steele, Amy Stubbs, Laurie Kemble, Danielle Beckham, Niccole Neal, Frank Lovecchio, Mary Mulrow, David Talan, Greg Moran, Anusha Krishnadasan, Kavitha Pathmarajah, Raquel Torrez, Eva Gonzalez, Gabina Martin, Noemi Quinteros Urzagaste, Jacklyn Furoy, Mayra Hernandez, Claire Collison, Andrea Dugas, Anna Duval, Raphaelle Beard, Ama Avornu, Rebecca Medina

**Affiliations:** ^1^ Department of Emergency Medicine Johns Hopkins University School of Medicine Baltimore MD USA; ^2^ Department of International Health Johns Hopkins University School of Public Health Baltimore MD USA

**Keywords:** co‐infection, emergency department, hospitalization, influenza, multiplex

## Abstract

**Background:**

Influenza causes significant morbidity and mortality in the United States. Among patients infected with influenza, the presence of bacterial co‐infection is associated with worse clinical outcomes; less is known regarding the clinical importance of viral co‐infections. The objective of this study was to determine rates of viral co‐infections in emergency department (ED) patients with confirmed influenza and association of co‐infection with disease severity.

**Methods:**

Secondary analysis of a biorepository and clinical database from a parent study where rapid influenza testing was implemented in four U.S. academic EDs, during the 2014–2015 influenza season. Patients were systematically tested for influenza virus using a validated clinical decision guideline. Demographic and clinical data were extracted from medical records; nasopharyngeal specimens from influenza‐positive patients were tested for viral co‐infections (ePlex, Genmark Diagnostics). Patterns of viral co‐infections were evaluated using chi‐square analysis. The association of viral co‐infection with hospital admission was assessed using univariate and multivariate regression.

**Results:**

The overall influenza A/B positivity rate was 18.1% (1071/5919). Of the 999 samples with ePlex results, the prevalence of viral co‐infections was 7.9% (79/999). The most common viral co‐infection was rhinovirus/enterovirus (RhV/EV), at 3.9% (39/999). The odds of hospital admission (OR 2.33, 95% CI: 1.01–5.34) increased significantly for those with viral co‐infections (other than RhV/EV) versus those with influenza A infection only.

**Conclusion:**

Presence of viral co‐infection (other than RhV/EV) in ED influenza A/B positive patients was independently associated with increased risk of hospital admission. Further research is needed to determine clinical utility of ED multiplex testing.

## 1 INTRODUCTION

Influenza infections are caused by influenza viruses with multiple circulating types, subtypes, and antigenic‐distinct strains and are responsible for up to 650,000 annual deaths worldwide, and 61,000 deaths in the United States annually since 2010.^1^, ^2^, ^3^ These infections pose a substantial burden on the healthcare system, given the significant annual morbidity and mortality. Certain groups of individuals are at greater risk for influenza‐related complications, including the elderly, the immunocompromised, and individuals with chronic co‐morbid conditions such as cardiovascular disease, cancer, diabetes, HIV/AIDS, and kidney disease.^1^

Previous studies have shown that in individuals infected with influenza viruses, rates of bacterial co‐infections are substantial, ranging between 11% and 35%.^4^, ^5^ The presence of bacterial co‐infection in those with influenza has also been widely reported to be associated with more complicated disease course, higher risk of intensive care unit (ICU) admission, and increased mortality.^4^, ^6^, ^7^ There has been relatively less research describing the overall burden and risks associated with viral co‐infection among those with influenza. Reported rates of influenza viral co‐infection from prior studies in varied settings range between 4% and 6%.^8^, ^9^, ^10^ There have been a few studies that report increased risk of severe illness in influenza‐infected patients found to have viral co‐infections, but these have principally been restricted to inpatients.^8^, ^11^

The recent developments and now widespread availability of multiplex viral testing platforms provide an opportunity to identify viral co‐infections in patients with influenza. We examined a large biorepository of nasopharyngeal (NP) specimens from a parent multi‐center emergency department (ED) study of patients who tested positive for either influenza A or B (influenza A/B). Our objective was to define rates, and demographic and clinical correlates of viral co‐infections in those with influenza A/B, as well as determine whether the presence of viral co‐infection was associated with more severe illness, using inpatient admission as a proxy for disease severity.

## 2 METHODS

### 2.1 Study design and data collection

We conducted a secondary analysis of a biorepository and clinical data set from a parent study, which was designed to assess the impact of systematic influenza testing on treatment for influenza in the ED.^12^ For the parent study, four participating sites (Johns Hopkins Hospital [JHH], Baltimore, MD; Truman Medical Center, Kansas City, MO; Maricopa Medical Center, Phoenix, AZ; Olive View‐UCLA Medical Center [OV‐UCLA‐Medical Center], Sylmar, CA) implemented a previously validated clinical decision guideline (CDG)^13^ coupled with electronic decision support to guide systematic influenza testing from ED triage. From November 1, 2014 to April 30, 2015, all adult ED patients age 18 or older were assessed at triage for presence of respiratory signs and/or symptoms to determine if they met testing criteria for influenza; those who did were tested with the GeneXpert assay (Cepheid, Sunnyvale, CA). Waste NP specimens were stored at −80°C for future use. A structured data collection form completed by trained research coordinators using the electronic medical record was used to collect demographic, clinical information (including comorbidities), and clinical outcome (including patient disposition).

### 2.2 Patient consent statement

Patient written consent was obtained. The parent influenza cohort (JHU IRB00041135, JHU IRB00141101) was approved by the JHU Institutional Review Boards and those of each participating institution for collection and analysis of specimens and clinical data; the biorepository analytic plan was approved by the JHU Institutional Review Board (JHU IRB00135664).

### 2.3 Specimen collection

**FIGURE 1:**
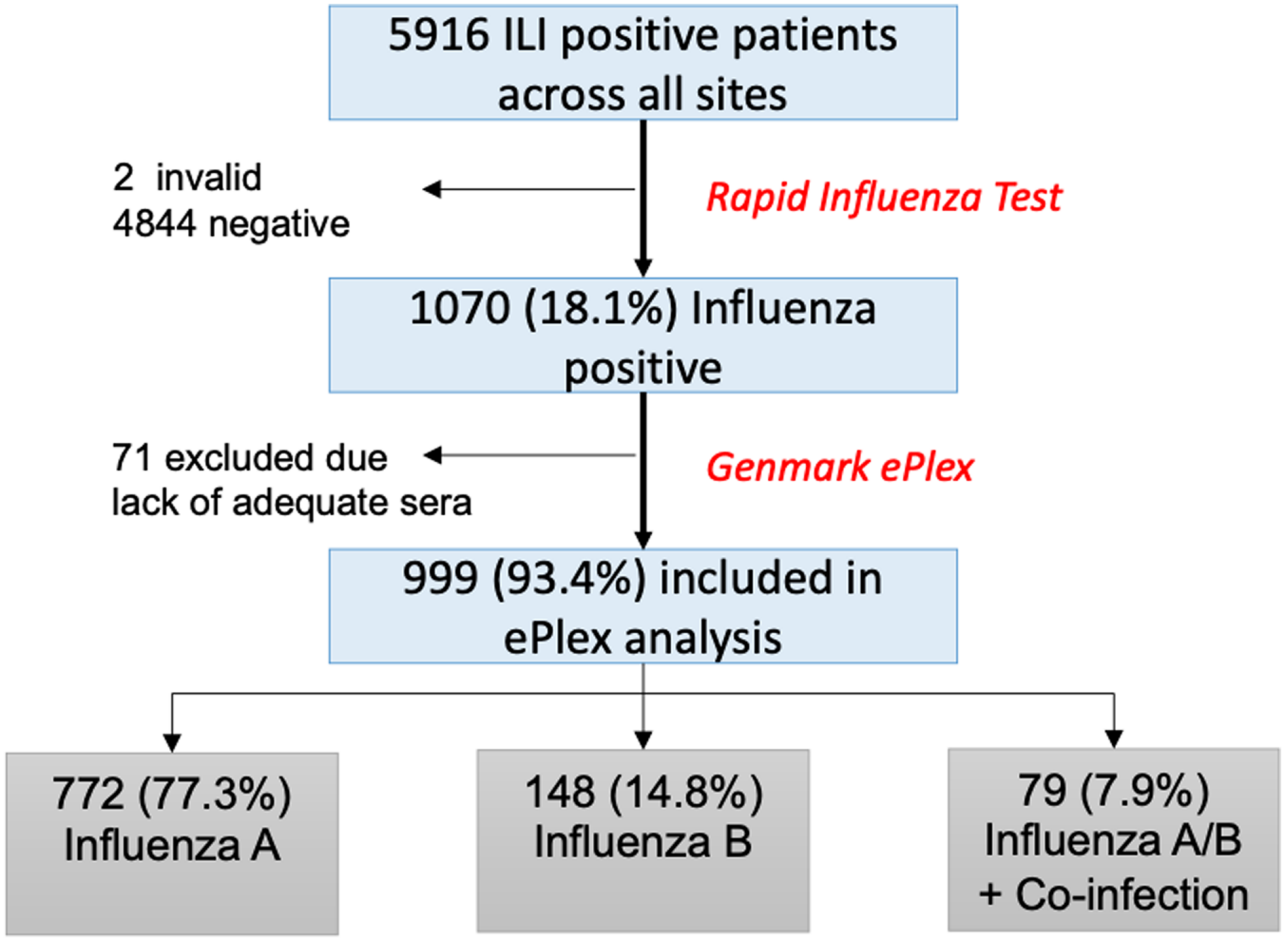
Schema of samples included for molecular detection

From the parent study, a total of 5916 patients across the four sites met the CDG criteria and were tested for influenza by the GeneXpert assay, of which 1070 tested positive for influenza. A total of 999 samples had adequate NP specimen available (>500 μl) allowing for further molecular detection using the ePlex RP RUO cartridges (Genmark Diagnostics, Carlsbad, CA) and were included for detailed analysis (Figure 1).

### 2.4 Molecular detection

Influenza positive NP specimens were evaluated utilizing ePlex RP RUO cartridges (Genmark Diagnostics, Carlsbad, CA). Testing was performed per manufacturer instructions. Briefly, 200 μl of the NP specimen was added to the sample delivery device, vortexed for 10 s and added to the RP RUO cartridge. Cartridges were loaded onto the ePlex, and the time to result was 1 h and 40 min. The RP RUO cartridges contain a test menu of the following: adenovirus (AdV); coronavirus HKU1, NL63, and OC43; MERS (CoV); human metapneumovirus (hMPV); influenza A, A/H1N1, A/H1N1pdm 2009, A/H3, and B; parainfluenza 1–4 (PIV); rhinovirus/enterovirus (RhV/EV); respiratory syncytial virus A/B (RSV); *Bordatella pertussis*; *Chlamydia pneumoniae*; *Legionella pneumophilla*; and *Mycoplasma pneumoniae*.

### 2.5 Outcomes and statistical analysis

Samples from patients who received rapid influenza testing were analyzed for basic demographic characteristics, hospitalization, ICU stay, and death. ePlex was utilized to determine influenza virus (IV) subtype and presence of viral co‐infection. For the purpose of analysis, we categorized patients into one of four groups, influenza A virus only (IAV); influenza B virus only (IBV), influenza A or B virus + RhV/EV co‐infection (IAV/IBV + RhV/EV); and influenza A or B virus + all other viral co‐infections excluding RhV/EV (IAV/IBV + non RhV/EV). We chose to categorize viral co‐infection cases as IAV/IBV with RhV/EV separately from all other viral co‐infections, given recent literature demonstrating an attenuating effect of RhV/EV on IAV.^14^, ^15^ Notably, IAV/IBV co‐infections with RhV/EV made up almost half of co‐infections observed in our population. Of note, there were two patients that tested positive for both IAV and IBV. As these did not fit in either IAV only or IBV only groups and given the small number, they were included in the IAV/IBV + non RhV/EV co‐infection group. The primary outcome of interest was hospital admission, which was used as a marker of disease severity. Chi‐squared analysis was used to determine the rates and patterns of viral‐co‐infections. Univariate analysis was conducted to determine the odds of admission based on presence of viral co‐infection; a multivariate logistic model was used to adjust for sex, age (categorical), and the presence or absence of certain underlying medical conditions (see Table 4), which would likely influence the clinical decision to admit (primary outcome).

## 3 RESULTS

A total of 5916 patients were tested for influenza across the four EDs. The majority of patients had only one ED encounter (5649/5916, 95.5%); 267 (4.5%) had two or more encounters (Table 1); 1070 of the 5916 patients (18.1%) were influenza positive (influenza A or B) by GeneXpert. Of these, 999 (93.4%) had adequate NP samples for advanced molecular detection with ePlex (Figure 1). The median age of participants was 45 years with an interquartile range (IQR) of 25 years (30–55 years). The majority of patients were female (3582/5916, 60.5%) across all study sites. “Chronic Lung Disease” was the most common identified comorbidity at 3 of the clinical sites (1756/5916, 29.7%) except for OV‐UCLA‐Medical Center, where “Metabolic/Endocrine Disorders” was the most common. Hospitalization rates ranged from 13.4% (OV‐UCLA Medical Center) to 29.5% (JHH). Overall, less than 2% (110/5916) of visits resulted in an ICU admission, with only 10 encounters (0.2%) resulting in death.

**TABLE 1:** Encounter characteristics by study site (N = 5916)

	Johns Hopkins Hospital	Maricopa Medical Center	OV‐UCLA Medical Center	Truman Medical Center	Total
Visits					
Number of visits	1696	1278	2016	926	5916
Visit 1	1604	1241	1911	893	5649
Visit 2	84	37	101	33	255
Visit 3	7	0	4	0	11
Visit 4	1	0	0	0	1
Demographics					
Age (%)					
18–29	496 (29.3)	398 (31.1)	343 (17.0)	222 (24.0)	1459 (24.7)
30–44	415 (24.5)	363 (28.4)	490 (24.3)	229 (24.7)	1497 (25.3)
45–59	498 (29.4)	360 (28.2)	739 (36.7)	364 (39.3)	1961 (33.1)
60–74	209 (12.3)	122 (9.5)	377 (18.7)	95 (10.3)	803 (13.6)
75+	78 (4.6)	35 (2.7)	67 (3.3)	16 (1.7)	196 (3.3)
Gender (%)					
Female	1039 (61.3)	772 (60.4)	1242 (61.6)	529 (57.1)	3582 (60.5)
Male	657 (38.7)	506 (39.6)	774 (38.4)	397 (42.9)	2334 (39.5)
Comorbidities					
Chronic lung disease	734 (43.3)	264 (20.7)	366 (18.2)	392 (42.3)	1756 (29.7)
Asthma	531 (31.3)	240 (18.8)	300 (14.9)	262 (28.3)	1333 (22.5)
COPD	207 (12.2)	38 (3.0)	53 (2.6)	169 (18.3)	467 (7.9)
Cardiovascular disease	315 (18.6)	103 (8.1)	134 (6.6)	165 (17.8)	717 (12.1)
CAD	128 (7.5)	39 (3.1)	61 (3.0)	82 (8.9)	310 (5.2)
CHF	155 (9.1)	33 (2.6)	26 (1.3)	64 (6.9)	278 (4.7)
Hematologic disease	79 (4.7)	10 (0.8)	16 (0.8)	10 (1.1)	115 (1.9)
Renal disease	157 (9.3)	49 (3.8)	46 (2.3)	69 (7.5)	321 (5.4)
Metabolic/endocrine disorders	385 (22.7)	239 (18.7)	458 (22.7)	233 (25.2)	1315 (22.2)
Diabetes	302 (17.8)	195 (15.3)	402 (19.9)	184 (19.9)	1083 (18.3)
Hepatic disease	195 (11.5)	65 (5.1)	41 (2.0)	102 (11.0)	403 (6.8)
Neurological disease	276 (16.3)	61 (4.8)	102 (5.1)	125 (13.5)	564 (9.5)
Cancer	148 (8.7)	25 (2.0)	82 (4.1)	40 (4.3)	295 (5.0)
Autoimmune disorder	83 (4.9)	14 (1.1)	78 (3.9)	31 (3.3)	206 (3.5)
HIV/AIDS	188 (11.1)	23 (1.8)	27 (1.3)	20 (2.2)	258 (4.4)
Level of care/death					
Hospitalized (%)	501 (29.5)	230 (18.0)	271 (13.4)	193 (20.8)	1195 (20.2)
ICU (%)	48 (2.8)	14 (1.1)	31 (1.5)	17 (1.8)	110 (1.9)
Death (%)	7 (0.4)	0 (0)	2 (0.1)	1 (0.1)	10 (0.2)
Rapid influenza testing outcomes					
Influenza A	273 (16.1)	180 (14.	288 (14.3)	138 (14.9)	879 (14.9)
Influenza B	50 (2.9)	22 (1.7)	80 (4.0)	39 (4.2)	191 (3.2)
Influenza negative	1373 (81.0)	1076 (84.2)	1646 (81.6)	749 (80.9)	4844 (81.9)
Invalid	0 (0.0)	0 (0.0)	2 (0.1)	0 (0.0)	2 (0.0)
Total	1696	1278	2016	926	5916

Abbreviations: CAD, coronary artery disease; CHF, congestive heart failure; CLD, chronic liver disease; COPD, chronic obstructive pulmonary disease; ICU, intensive care unit.

Rapid testing revealed IAV in 14.9% (879/5916) of cases and IBV in 3.2% (191/5916) of cases, giving an overall IAV/IBV positive rate of 18.1% (1070/5916); with 81.9% (4844/5916) of specimens testing influenza negative. No IAV plus IBV co‐infection was noted via rapid test. When comparing those with positive influenza results via rapid test with those with a negative or indeterminate test, we did not see a substantive difference in mean age (influenza negative/indeterminate: 43.8 [SD = 15.7] years; influenza positive: 43.6 [SD = 17.1] years) or gender (influenza negative/indeterminate: 2916 [60.2%] female, influenza positive: 666 [62.2%] female). The presence of any of the listed comorbidities was slightly higher in those without positive rapid influenza tests (influenza negative/indeterminate: 2826 [58.3%], influenza positive: 577 [53.9%], chi‐squared *P* value = 0.009).

Further characterization by Genmark ePlex testing of influenza subtypes and presence of viral co‐infections for the 999 specimens available for detailed characterization is described in Table 2. IAV was the most common virus identified (772/999, 77.3%), and IBV was the second most commonly observed (148/999, 14.8%). The most dominant subtype of IVs was influenza A/H3 virus, accounting for 76.3% of samples (762/999). The most commonly observed viral co‐infection was IAV with RhV/EV (38/999, 3.8%). Other non RhV/EV co‐infections identified with IAV were CoV 229E, NL63, and OC3 (15/999, 1.5%) and AdV, PIV, RSV, and hMPV (19/999, 1.9%), whereas IBV plus other non RhV/EV co‐infection rates were much lower (4/999, 0.4%), and these included AdV, PIV and RSV. Of note, 6 specimens initially identified as IAV by rapid Xpert testing were reported as IBV by ePlex testing, and 4 specimens initially identified as IBV by Xpert testing were reported to be influenza A/H3 virus by ePlex. Two specimens noted to be IAV on Xpert testing were found to have IAV plus IBV co‐infections by ePlex testing. The ePlex cartridges used in the study include bacteria; there was only 1 specimen positive for influenza and a bacterial co‐infection. Of note, there were no significant differences in age and gender for those excluded (N = 71) versus those included who underwent detailed molecular characterization (N = 999). The mean age of those excluded versus included was 43.8 (SD = 17.3) and 41.7 (SD = 13.9), respectively. The proportion of female patients excluded versus included was 65% and 62%, respectively.

**TABLE 2:** Summary of ePlex results by site (N = 999)

	Johns Hopkins Hospital	Maricopa Medical Center	OV‐UCLA Medical Center	Truman Medical Center	Total
Virus					
Influenza A	3 (1.0)	0 (0.0)	5 (1.5)	1 (0.6)	9 (0.9)
Influenza A/H1N1 pdm 2009	0 (0.0)	0 (0.0)	1 (0.3)	0 (0.0)	1 (0.1)
Influenza A/H3^a^	237 (76.0)	156 (80.8)	240 (75.7)	129 (72.9)	762 (76.3)
Influenza B	48 (15)	23 (11.9)	42 (13.2)	35 (19.8)	148 (14.8)
Influenza A/RhV/EV	14 (4.5)	7 (3.6)	13 (4.1)	4 (2.3)	38 (3.8)
Influenza B/RhV/EV	1 (0.3)	0 (0.0)	0 (0.0)	0 (0.0)	1 (0.1)
Influenza A/H3/coronavirus^b^	2 (0.6)	5 (2.6)	8 (2.5)	0 (0.0)	15 (1.5)
Influenza A/H3/influenza B	0 (0.0)	0 (0.0)	2 (0.6)	0 (0.0)	2 (0.2)
Influenza A or A/H3/other^c^	7 (2.2)	2 (1.0)	6 (1.9)	4 (2.3)	19 (1.9)
Influenza B/other^d^	0 (0.0)	0 (0.0)	0 (0.0)	4 (2.3)	4 (0.4)
Total	312	193	317	177	999

^a^ Presumptive H3N2.

^b^ Coronavirus 229E, coronavirus NL63, coronavirus OC43.

^c^ Adenovirus, Metapneumovirus, *Mycoplasma pneumoniae*, Parainfluenza 2, Parainfluenza 4, respiratory syncytial virus A, respiratory syncytial virus B.

^d^ Adenovirus, parainfluenza, respiratory syncytial virus B.

Patient characteristics by type of co‐infection are summarized in Table 3 with the following four categories, based on co‐infection status: IAV only, IBV only, IAV/IBV + RhV/EV, and IAV/IBV + non‐RhV/EV viral co‐infection (this group included the two patients that were positive for both IAV and IBV). With regard to differences observed in age based on co‐infection status, a higher proportion of those with IAV/IBV + non‐RhV/EV viral co‐infection were >60 years versus those in the other three categories; a significantly high proportion of those with IAV/IBV + RhV/EV were in the 18–29 year age group, compared with those in the other categories. We also found that patients in the IAV/IBV + non‐RhV/EV viral co‐infection were significantly more likely to have HIV/AIDS (6/40, 15%; *P* < 0.05) and cancer (4/40, 10%; *P* = 0.07) and require hospitalization (12/40, 30%; *P* = 0.02) when compared with the other categories.

**TABLE 3:** Patient characteristics by type of influenza co‐infection as determined by ePlex (N = 999)

	Influenza A virus only (%)	Influenza B virus only (%)	Influenza A/B virus + RhV/EV co‐infection (%)	Influenza A/B virus + non RhV/EV co‐infection (%)	Total	Chi‐squared (*P* value)
Hospital site						
JHH	240 (31.1)	48 (32.4)	15 (38.5)	9 (22.5)	312 (31.2)	9.26 (0.41)
MMC	156 (20.2)	23 (15.5)	7 (17.9)	7 (17.5)	193 (19.3)	
OV‐UCLA	246 (31.9)	42 (28.4)	13 (33.3)	16 (40.0)	317 (31.7)	
TMC	130 (16.8)	35 (23.6)	4 (10.3)	8 (20.0)	177 (17.7)	
Demographics						
Age (%)						
18–29	207 (26.8)	41 (27.7)	20 (51.3)	9 (22.5)	277 (27.7)	24.47 (0.02)**
30–44	190 (24.6)	47 (31.8)	4 (10.3)	11 (27.5)	252 (25.2)	
45–59	217 (28.1)	37 (25.0)	10 (25.6)	11 (27.5)	275 (27.5)	
60–74	110 (14.2)	20 (13.5)	4 (10.3)	9 (22.5)	143 (14.3)	
75+	48 (6.2)	3 (2.0)	1 (2.6)	0 (0.0)	52 (5.2)	
Gender (%)						
Female	478 (61.9)	89 (60.1)	23 (59.0)	30 (75.0)	620 (62.1)	3.24 (0.36)
Male	294 (38.1)	59 (39.9)	16 (41.0)	10 (25.0)	379 (37.9)	
Comorbidities						
Chronic lung disease	214 (27.7)	41 (27.7)	11 (28.2)	10 (25.0)	276 (27.6)	0.15 (0.99)
Asthma	174 (22.5)	32 (21.6)	10 (25.6)	9 (22.5)	225 (22.5)	0.29 (0.96)
COPD	49 (6.3)	11 (7.4)	2 (5.1)	4 (10.0)	66 (6.6)	1.13 (0.77)
Cardiovascular disease	92 (11.9)	12 (8.1)	5 (12.8)	7 (17.5)	116 (11.6)	3.24 (0.36)
CAD	39 (5.1)	3 (2.0)	3 (7.7)	4 (10.0)	49 (4.9)	5.54 (0.14)
CHF	34 (4.4)	8 (5.4)	1 (2.6)	3 (7.5)	46 (4.6)	1.42 (0.70)
Hematologic disease	14 (1.8)	1 (0.7)	0 (0.0)	0 (0.0)	15 (1.5)	2.39 (0.49)
Renal disease	43 (5.6)	7 (4.7)	0 (0.0)	4 (10.0)	54 (5.4)	4.05 (0.26)
Metabolic/endocrine disorders	182 (23.6)	25 (16.9)	5 (12.8)	11 (27.5)	223 (22.3)	5.86 (0.12)
Diabetes	151 (19.6)	22 (14.9)	4 (10.3)	8 (20.0)	185 (18.5)	3.69 (0.30)
Hepatic disease	31 (4.0)	6 (4.1)	1 (2.6)	2 (5.0)	40 (4.0)	0.31 (0.96)
Neurological disease	74 (9.6)	7 (4.7)	1 (2.6)	2 (5.0)	84 (8.4)	6.32 (0.10)*
Cancer	40 (5.2)	2 (1.4)	3 (7.7)	4 (10.0)	49 (4.9)	7.01 (0.07)*
Autoimmune disorder	25 (3.2)	4 (2.7)	1 (2.6)	2 (5.0)	32 (3.2)	0.59 (0.90)
HIV/AIDS	31 (4.0)	5 (3.4)	1 (2.6)	6 (15.0)	43 (4.3)	11.86 (0.008)**
Level of care or death						
Hospitalized (%)	144 (18.7)	16 (10.8)	6 (15.4)	12 (30.0)	178 (17.8)	9.54 (0.02)**
ICU (%)	14 (1.8)	1 (0.7)	0 (0.0)	2 (5.0)	17 (1.7)	4.27 (0.52)
Death (%)	0 (0.0)	0 (0.0)	0 (0.0)	0 (0.0)	0 (0.0)	‐
Total	772	148	39	40	999	

Abbreviations: CAD, coronary artery disease; CHF, congestive heart failure; CLD, chronic liver disease; COPD, chronic obstructive pulmonary disease; ICU, intensive care unit.

* *P* value is >0.05–0.1.

** *P* value is <0.05.

Associations were further quantified using univariate and multivariable analysis (Table 4). In univariate analysis when compared with patients with IAV only, patients with IBV only were significantly less likely to be admitted (OR 0.53, 95% CI: 0.30–0.92), whereas patients with IAV/IBV + non‐RhV/EV viral co‐infection showed a trend towards higher likelihood of admission (OR 1.87, 95% CI: 0.93–3.76). In the multivariable model, adjusting for age, gender, cancer, HIV/AIDS, coronary artery disease (CAD), diabetes, congestive heart failure (CHF), chronic liver disease (CLD), neurologic problems, and hospital site, we found that patients with influenza A/B + non‐RhV/EV viral co‐infection were over twice as likely to be admitted when compared with those with influenza A only (OR 2.33, 95% CI: 1.01–5.34; *P* = 0.046). However, this association was absent for those with influenza A/B + RhV/EV (OR 1.20, 95% CI: 0.43–3.32; *P* = 0.724). The comorbidities in the multivariate model were chosen due to their frequency of association with influenza infection and admission. In the multivariable model, those with IBV infections showed a trend towards less hospitalized admissions compared with those infected with IAV, although this effect was somewhat diminished in the multivariate model (OR 0.60, 95% CI: 0.33–1.10; *P* = 0.100).

**TABLE 4:** Regression model of factors affecting hospital admission (N = 999)

	Single covariate regression		Multiple logistic regression model controlling for age & gender		Full multiple logistic regression model	
	OR (95% CI)	*P* value	OR (95% CI)	*P* value	OR (95% CI)	*P* value
Influenza type						
Influenza A virus	‐	‐	‐	‐	‐	‐
Influenza B virus	0.53 (0.30–0.92)	0.023**	0.60 (0.34–1.07)	0.084*	0.60 (0.33–1.10)	0.100
Influenza/RhV/EV	0.79 (0.33–1.93)	0.609	1.08(0.41–2.80)	0.882	1.20 (0.43–3.32)	0.724
Influenza/nonRhV/EV	1.87 (0.93–3.76)	0.080*	2.19 (1.04–4.62)	0.039**	2.33 (1.01–5.34)	0.046**
Hospital site						
JHH	‐	‐	‐	‐	‐	‐
MMC	0.40 (0.24–0.65)	<0.001***	0.42 (0.25–0.71)	0.001***	0.75 (0.42–1.32)	0.316
OV‐UCLA	0.32 (0.21–0.50)	<0.001***	0.24 (0.15–0.38)	<0.001***	0.47 (0.28–0.80)	0.006***
TMC	0.63 (0.40–1.00)	0.048	0.69 (0.42–1.12)	0.130	0.86 (0.50–1.48)	0.587
Demographics						
Age (%)						
18–29	‐	‐	‐	‐	‐	‐
30–44	2.35 (1.21–4.57)	0.012**	2.34 (1.20–4.56)	0.012**	2.41 (1.20–4.86)	0.014**
45–59	5.47 (2.98–10.05)	<0.001***	5.48 (2.98–10.06)	<0.001***	5.08 (2.62–9.85)	<0.001***
60–74	8.35 (4.38–15.91)	<0.001***	8.26 (4.33–15.75)	<0.001***	7.11 (3.41–14.81)	<0.001***
75+	25.62 (11.86–55.33)	<0.001***	25.53 (11.81–55.15)	<0.001***	19.11 (7.83–46.59)	<0.001***
Gender (%)						
Female	‐	‐	‐		‐	‐
Male	1.20 (0.87–1.68)	0.271	1.15 (0.81–1.64)	0.429	1.28 (0.86–1.89)	0.222
Comorbidities						
CLD	3.29 (2.35–4.61)	<0.001***	3.84 (2.65–5.56)	<0.001***	2.84 (1.89–4.26)	<0.001***
CAD	7.79 (4.29–14.16)	<0.001***	3.59 (1.89–6.82)	<0.001***	1.92 (0.93–4.00)	0.079*
CHF	7.55 (4.09–13.93)	<0.001***	5.07 (2.65–9.70)	<0.001***	2.19 (1.05–4.55)	0.036**
Diabetes	3.25 (1.26–4.67)	<0.001***	1.93 (1.30–2.86)	0.001***	1.54 (0.99–2.38)	0.053*
Cancer	3.15 (1.73–5.74)	<0.001***	1.40 (0.73–2.68)	0.315	0.98 (0.47–2.05)	0.958
HIV/AIDS	3.58 (1.91–6.73)	<0.001***	4.00 (2.04–7.84)	<0.001***	2.26 (1.05–4.85)	0.036**
Neurologic	4.32 (2.71–6.89)	<0.001***	3.17 (1.90–5.30)	<0.001***	2.16 (1.21–3.85)	0.009***

Abbreviations: CAD, coronary artery disease; CHF, congestive heart failure; CLD, chronic liver disease.

* *P* value is 0.05 to <0.1.

** *P* value is 0.01–0.05.

*** *P* value is <0.01.

Of note, when the influenza A/B plus RhV/EV and influenza A/B plus other viral co‐infection groups were pooled (i.e., influenza A/B + ANY co‐infection), we found that ANY viral co‐infection compared with influenza A only resulted in an observed OR of 1.75 (95% CI: 0.90–3.39; *P* = 0.097). Thus, including RhV/EV in the co‐infection category diminishes the association between co‐infection and admission.

## 4 DISCUSSION

Overall, we observed that in ED patients with influenza A or B virus, viral co‐infections in our study occurred in 7.9% of patients, and patients with certain viral co‐infections were more than twice as likely to get admitted when controlling for age and comorbidities known to commonly influence a clinician's decision to admit. We observed that in patients infected with influenza A or B virus, RhV/EV was the most common viral co‐infection observed, consistent with what has been observed in other recent studies.^8^, ^10^ We also noted that patients with IAV/IBV + RhV/EV co‐infection were relatively less likely to require admission (versus those with any other viral co‐infection) suggesting a lower severity of illness among that group, with similar admission rates to those with IAV infections alone. One theory to explain this phenomenon was noted in experimental animal studies that have established that infection with RhV effectively attenuates disease after infection with influenza A, by inducing rapid clearance from the lungs. In humans, a study of 496 patients with H1N1 showed that patients infected with influenza and RhV had lower rates of admission to the hospital (52.6% vs. 69.8%) and decreased rates of oxygen use (21.1% vs. 31.0%), compared with patients without co‐infection; however, this association was not statistically significant.^14^ Additionally, a recent study by Wu et al demonstrated that infection with RhV or EV may be protective against influenza A infections.^15^ Notably, one prior study by Drew et al demonstrated that in patients infected with influenza A/B virus plus another viral co‐infection (excluding RhV/EV), there was an increased risk of hospital admission, consistent with our findings here, although that was not specific to an ED population.^16^ As noted, although we did not see worsening infection in our cohort study with IAV/IBV + RhV/EV co‐infection, we did not appreciate any protective impact in this study either.

Multiplex upper respiratory viral testing has been described in the literature as a potential approach to aid in antibiotic stewardship.^17^, ^18^, ^19^ In a pediatric randomized control trial led by Rao et al, with respiratory viral panel (RVP) testing as an intervention, there was no impact of testing availability on antibiotic prescribing (RR, 1.1, 95% CI: 0.9–1.4).^20^ However, in an adult study of 2000 patients, availability of rapid PCR testing resulted in change in management occurring in 58% of the cases resulting in a decrease of inappropriate/unsupported antibiotic and anti‐viral prescribing by 24.5% and 9%, respectively. This resulted in a projected cost savings of >$578,000 due to deferred admissions and reduction in antiviral prescribing at the study site.^21^ While the impact on admission and prescribing is well documented, the impact of point‐of‐care influenza testing on ED‐length of stay is unknown.^22^

In our study, we sought to specifically describe the impact of multiplex testing to identify potential co‐infections early. Our study found higher rates of hospital admission in those with viral co‐infections (other than RhV/EV), which may be marker for more severe disease, similar to what has been reported in previous studies,^8^, ^11^ particularly in high‐risk populations. A Zambian study identified that there were few differences in presenting symptoms between those with single‐pathogen infections and those with respiratory co‐infections. However, they did note headache was associated with co‐infection with any pathogen (age‐adjusted prevalence ratio [adj PR] 3.67, 95% CI: 1.36–9.88) and among participants with a diagnosed RSV co‐infection a longer duration of ILI symptoms (adj PR per additional day of symptoms 1.35, 95% CI: 1.02–1.78) and diarrhea (adj PR 3.23, 95% CI: 1.31–7.96).^23^ A study by Yang et al of 270 participants demonstrated that FilmArray Respiratory Panel testing can be successfully implemented in the ED and was useful not only to identify co‐infection (bacterial and viral) but also demonstrated that results of the FilmArray testing had a direct impact on antibiotic and antiviral testing (*P* < 0.001).^24^

Numerous diagnostic assays exist to determine co‐infections, including multiplex nucleic acid amplification and microarray‐based assays. A new challenge is understanding the performance evaluation of different assays and testing phase requirements.^20^, ^22^ A study by Diaz‐Decaro, which focuses on FDA‐approved respiratory multiplex assays for public health surveillance, identifies rhinovirus and enteroviruses as problematic targets for multiplex due to genetic homology and primer similarity increasing the risk of cross‐amplification and interference during multiplex testing.^22^ The small sample size of our study however prohibited us from distinguishing the impact of rhinovirus co‐infection versus enterovirus co‐infection. Larger prospective multisite observational study that includes adults as well as pediatric patients is required to understand the true role of viral co‐infection in clinical outcomes, and whether knowing the specific type of viral co‐infection is useful for prognostication and should be utilized to aid patient care decisions.

### 4.1 Limitations

There were several limitations to this study. First, despite evaluating a large number of influenza‐positive samples, the total number of non‐influenza respiratory viruses and co‐infected cases identified was relatively modest, limiting the ability to assess other potential outcomes, such as the association of co‐infection with ICU admission and/or mortality. Second, this study was restricted to adult ED patients and included only one influenza season, limiting generalizability. Third, clinical data were gathered retrospectively by chart review, and certain comorbidities may not have been fully captured. Fourth, there may have been cases of co‐infections that were missed or overcalled; there were 12 cases where the influenza type results from GenXpert and ePlex platform did not match. While that number was relatively small (<2% of cases overall) and might at least in part be explained by co‐infections, it is possible that one or both assays had false positive or false negative result. Finally, we used hospital admission as a proxy for severity of illness. However, a clinician's decision to admit may not be strictly influenced by disease severity and there are other factors not captured here (e.g. psychosocial) that may affect the admission decision, independent of the influenza infection itself. In our regression analysis, however, we control for a variety of potential confounders including demographic, multiple comorbidities, and hospital site. Future studies could prospectively assess clinician's reason for admission and/or a numerical scoring system for severity of illness (such as MEWS score) at the time of admission, to provide more unbiased measure of severity of illness.^25^

## 5 CONCLUSION

In conclusion, we found that the presence of viral co‐infection (other than RhV/EV) in ED influenza A/B positive patients was independently associated with increased risk of hospital admission. Our data suggest that multiplex testing may be valuable in practice when used to test higher risk populations or incorporated into a CDG for those patients found to have influenza A/B viral infection and could aid clinicians in predicting patient trajectory and helping with triage decisions for discharge or admission. The routine use of multiplex testing in the ED as a predictor for patient outcomes still requires more research.

## AUTHOR CONTRIBUTIONS

**Kerry Shannon:** Conceptualization; data curation; formal analysis. **Valerie Osula:** Conceptualization; project administration; manuscript preparation. **Kathryn Shaw‐Saliba:** Data curation; investigation. **Justin Hardick:** Data curation; investigation; methodology. **Breana McBryde:** Data curation; investigation. **Andrea Dugas:** Contributed to project administration and manuscript editing in her private capacity, the views expressed in this article do not represent the views of or endorsement by the United States Government or the FDA. **Yu‐Hsiang Hsieh:** Manuscript preparation. **Bhakti Hansoti:** Conceptualization; manuscript editing. **Richard Rothman:** Conceptualization; project administration; manuscript editing.

### PEER REVIEW

The peer review history for this article is available at https://publons.com/publon/10.1111/irv.12967.

## Data Availability

The de‐identified data that support the findings of this study are available from the corresponding author upon reasonable request.
